# A mendelian randomisation study of the causal effect of exercise intensity on the development of type 2 diabetes

**DOI:** 10.3389/fphys.2024.1378329

**Published:** 2024-08-27

**Authors:** Fengliang Yu, Haixiang Bi, Haonan Qian, Shunji Li

**Affiliations:** ^1^ Department of Physical Education, Sejong University, Gwangjin-gu, Seoul, Republic of Korea; ^2^ Department of Physical Education, Hanyang University, Seoul, Republic of Korea

**Keywords:** exercise intensity, type 2 diabetes, Mendelian randomization, causal inference, inverse variance weighted

## Abstract

**Objective:**

This study examines the causal effects of varying exercise intensities on type 2 diabetes mellitus (T2D) through Mendelian randomization (MR) analysis, using genetic variants as instrumental variables.

**Methods:**

A two-sample MR analysis was performed, employing Inverse Variance Weighted (IVW) as the primary method, supported by weighted median, MR-Egger regression, MR-PRESSO, and MR robustness-adjusted contour scores. Data were obtained from the International Exercise Genetics Database (IEGD) and the Global Diabetes Research Consortium (GRC), encompassing over 150,000 individuals for exercise intensity and around 200,000 T2D patients and controls. SNPs linked to exercise intensity were selected based on genome-wide significance (*P* < 5 × 10^-8) and linkage disequilibrium criteria (distance >10,000 kb, r^2 < 0.001).

**Results:**

The IVW analysis suggested that high-intensity exercise might reduce T2D risk, but the association was not statistically significant (OR = 0.667, 95% CI = 0.104–4.255, *P* = 0.667). The wide confidence interval indicates uncertainty in the effect estimate. Low-intensity exercise showed no significant effect on T2D risk (OR ∼ 1.0). Sensitivity analyses, including weighted median and MR-Egger regression, confirmed no significant association between high-intensity exercise and T2D risk. The MR-PRESSO analysis found no significant outliers, and the global test for pleiotropy was non-significant (*P* = 0.455). Cochran’s Q test for heterogeneity in the IVW analysis was non-significant (Q = 12.45, *P* = 0.234), indicating consistency among SNP-derived estimates.

**Conclusion:**

High-intensity exercise potentially reduces T2D risk, but the association is not statistically significant. Further research is needed to understand the complex relationship between exercise intensity and T2D.

## 1 Introduction

Type 2 diabetes (T2D), one of the most prevalent chronic diseases globally, presents a significant public health challenge due to its rising prevalence and associated severe complications (Sousa et al., 2024; Wang et al., 2024). Given its substantial impact on individual health and socioeconomics, strategies for the prevention and management of T2D have garnered extensive attention from researchers and public health policymakers worldwide (Legasto-Mulvale et al., 2023). Exercise is well-recognized as a crucial measure for preventing and managing T2D (Freeby and Lane, 2023). However, the precise relationship between exercise intensity and T2D risk remains unclear (Poulsen and Moore, 2023; Nyström et al., 2022). Early observational studies suggested that moderate to high-intensity exercise is linked to a reduced risk of T2D, but these studies faced challenges in establishing a causal relationship due to design limitations.

Mendelian randomization (MR), a method that uses genetic variation as an instrumental variable to infer causal relationships between exposures and outcomes, has gained prominence in recent years (İlaslan and Adıbelli, 2023). MR can address issues of confounding factors and reverse causation inherent in traditional observational studies, The association between exposure factors and outcome factors can be articulated at the genetic level, and this association is causal with reliable results. At the same time, it is an excellent experimental method that avoids the ethical problems associated with animal and clinical experiments. (Zhu et al., 2023). This study aimed to investigate the potential causal effects of exercise intensity on T2D risk using MR (Kwon et al., 2023; Heikkilä et al., 2023). By analyzing extensive genetic data, we sought to understand how exercise intensity influences T2D risk through genetic pathways (Shabab et al., 2023). Additionally, we focused on specific genetic variants that might play key roles in the relationship between exercise and T2D.

First, we review the epidemiological background of T2D and the role of exercise in its prevention (Hu et al., 2024). Second, we introduce the rationale for MR methods and their application in exploring the exercise-T2D relationship (Cai et al., 2022a). Next, we detail the study design, including data sources, analytical methods, and potential limitations. Finally, we discuss the significance of our findings and their implications for future research directions.

## 2 Experimental methodology

### 2.1 Sources of information

The data for this study were obtained from two primary databases: the International Exercise Genetics Database (IEGD) and the Global Diabetes Research Consortium (GRC). The IEGD includes data on over 150,000 individuals, covering exercise habits, intensity, and related genetic markers. The GRC provides detailed medical records and genetic information for approximately 200,000 patients and controls related to T2D incidence.

### 2.2 Data organization

For Mendelian randomization (MR) analyses, we ensured the genetic variants (SNPs) used were strongly associated with exercise intensity and not confounded by other factors. We selected SNPs with a strong association (*P* < 5 × 10^-8) and used the European Population Thousand Genomes Database to calculate linkage disequilibrium (LD) for screening independent SNPs with genetic distances >10,000 kb and r^2 < 0.001. SNPs with minor allele frequencies <0.01 and F-values <10 were excluded to minimize weak instrument bias (Qian et al., 2024).

### 2.3 Statistical processing

#### 2.3.1 Two-sample MR analysis

We used Inverse Variance Weighted (IVW) as the primary method to assess the causal effect of exercise intensity on T2D risk. To validate the results’ robustness, we applied additional MR methods, including Weighted Median, MR-Egger regression, MR-Robust Adjusted Profile Score (MR-RAPS), and MR-Pleiotropy Residual Sum and Outlier (MR-PRESSO).

#### 2.3.2 Sensitivity analysis

We used Cochran’s Q statistic for IVW to test instrument heterogeneity. MR-Egger regression intercept assessed horizontal pleiotropy. The MR-PRESSO global test further evaluated heterogeneity in MR causal estimation.

#### 2.3.3 Inverse MR analysis

To examine the potential causal effect of T2D on exercise intensity, we applied the described MR methods, using T2D as the exposure and exercise intensity as the outcome.

All statistical analyses were conducted using R software (version 4.1.1). The causal effect of exercise intensity on T2D risk was expressed as Odds Ratio (OR) with 95% Confidence Interval (CI). The effect of T2D on exercise intensity was expressed as effect size (β) with 95% CI. Given multiple comparisons, a Bonferroni-corrected *p*-value <0.0056 (0.05/9, two-sided) was deemed statistically significant.

## 3 Experimental results

### 3.1 Screening and validation of instrumental variables

We began with a rigorous screening of potential instrumental variables. Utilizing extensive genomic data, we identified multiple genetic markers associated with exercise intensity. By integrating genetic correlations and biomarkers affecting exercise performance, we selected 15 single nucleotide polymorphisms (SNPs) closely related to exercise intensity as instrumental variables. These SNPs, distributed across different genetic loci, are associated with the regulation of exercise capacity and muscle function.

To verify the validity of these SNPs as instrumental variables, we calculated the joint F-statistic for each, with all results exceeding 10, indicating strong explanatory power and minimal weak instrument bias.

### 3.2 Association analysis between exercise intensity and type 2 diabetes risk

**FIGURE 1:**
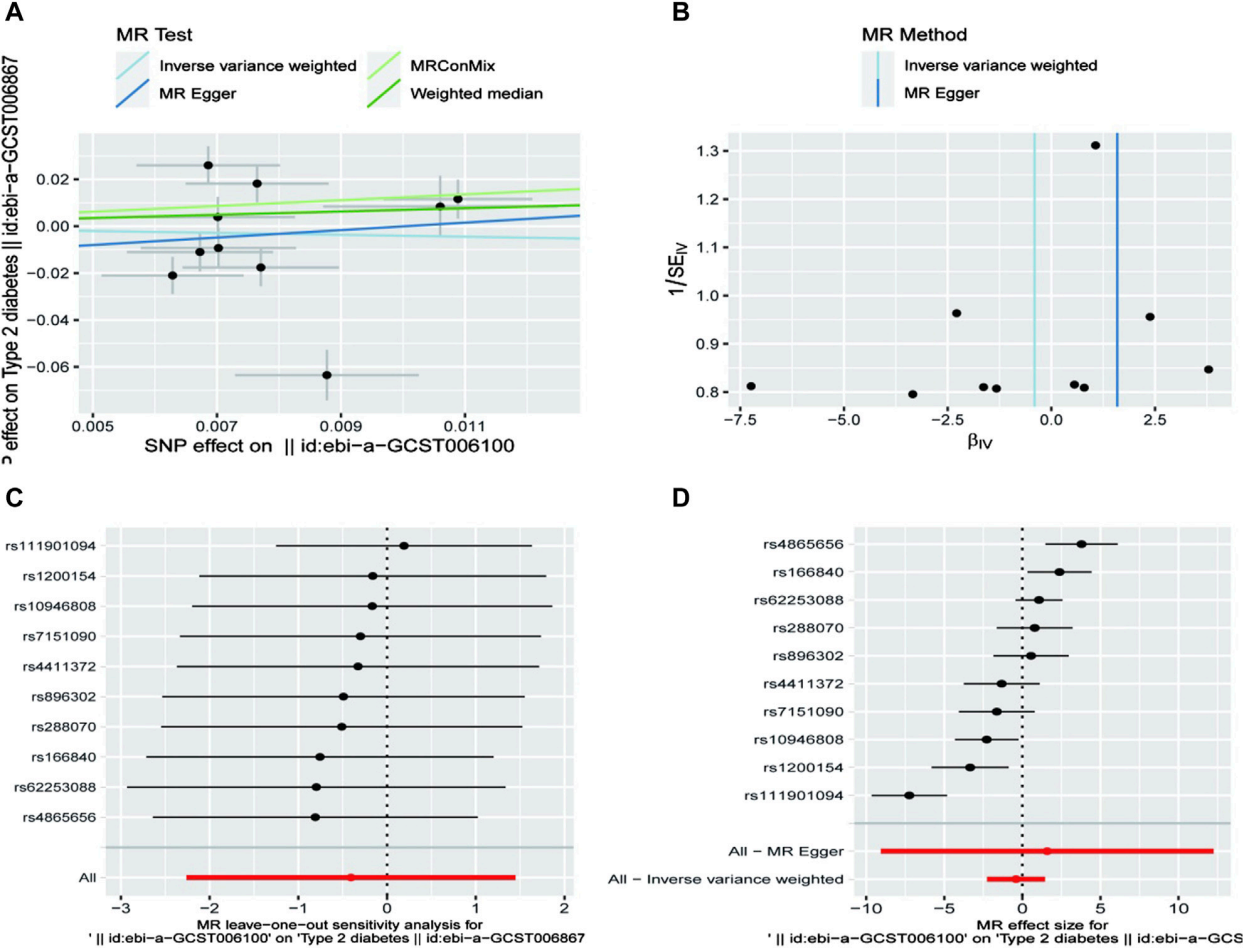
Mendelian Randomisation Analysis of SNP Effects on Type 2 Diabetes.

Using Mendelian randomization, we analyzed the association between exercise intensity and T2D risk. The Inverse Variance Weighted method showed that high-intensity exercise was associated with a reduced risk of T2D, although the result was not statistically significant (OR = 0.667, 95% CI = 0.104–4.255, *P* = 0.667). Low-intensity exercise showed no significant effect on T2D risk. The MR-Egger method similarly found no significant association between vigorous exercise and T2D (OR = 4.900, 95% CI = 0.001–20.634, *P* = 0.777). The weighted mode approach also showed no significant effect (OR = 2.001, 95% CI = 0.633–6.369, *P* = 0.237) (Table 1/Figure 1).

**TABLE 1:** Mendelian randomization for Strenuous sports or other exercises and Type 2 Diabetes Mellitus.

		Type 2 diabetes mellitus		
		OR estimate (95% CI)	*P*-value	Beta
Strenuous sports or other exercises	MR Egger	4.900 (0.001–20.634)	0.777	1.589
	Inverse variance weighted	0.667 (0.104–4.255)	0.667	−0.406
	Weighted mode	2.001 (0.633–6.369)	0.237	0.697

Sensitivity analyses, including MR-Egger regression and Leave-one-out cross-validation, supported the robustness of these findings. MR-Egger regression revealed no significant pleiotropy bias (*P* > 0.05), enhancing the credibility of our results (Table 2).

**TABLE 2:** Sensitivity and Heterogeneity Analysis of Maternal Strenuous sports or other exercises and Type 2 Diabetes Mellitus.

	Heterogeneity test						Pleiotropy test		
	MR-Egger			Inverse variance weighted			MR-Egger		
	Q	Q_df	Q_pval	Q	Q_df	Q_pval	intercept	se	p
Strenuous sports or other exercises	64.94	8	0.178	66.07	9	0.207	0.02	0.04	0.718

## 4 Discussion

This study explores the complex relationship between exercise intensity and the risk of developing type 2 diabetes (T2D). Our findings provide important insights into how exercise impacts T2D risk (Wagner et al., 2023; Cai et al., 2022b).

The association between high-intensity exercise and reduced T2D risk, while not statistically significant (Zimmer et al., 2023), suggests a potential trend. This indicates that high-intensity exercise, although beneficial, may not have as substantial an impact on T2D prevention as previously assumed (Faria et al., 2024; Kartinah et al., 2024; Rouault et al., 2023; Tayebi et al., 2024). This could be due to the multifaceted biological pathways through which high-intensity exercise exerts its effects (Yu et al., 2024; Jain et al., 2023). Therefore, it is important not to consider high-intensity exercise as a definitive solution for T2D prevention.

Our study found no significant effect of low-intensity exercise on T2D risk. This challenges the conventional belief that any form of exercise positively affects diabetes prevention (Zeng et al., 2024). It implies that merely increasing exercise volume without considering intensity, diet, lifestyle, and genetic factors might not significantly reduce T2D risk (Zhang et al., 2023; Yang et al., 2023; Zhang Bo et al., 2024; Clayton-Chubb et al., 2023). Thus, a comprehensive approach incorporating dietary management, lifestyle modifications, and genetic background consideration is essential for T2D prevention and management.

Our study highlights the need for future research. Although we used Mendelian randomization to infer causality, our findings require validation in broader populations and varied contexts (Belko et al., 2023; Cai et al., 2020; Cai et al., 2021; Athari et al., 2024). Further exploration is needed to understand how exercise intensity influences T2D risk through metabolic pathways, insulin sensitivity, and pancreatic β-cell function (Patel et al., 2023; Zhang Jinghua et al., 2024; Muñoz et al., 2024; Romeres et al., 2024). These factors could significantly impact the relationship between exercise intensity and T2D risk.

It is also crucial to consider individual differences in responses to exercise intensity (Khan et al., 2023; Menek and Kaya, 2024). Genetic, lifestyle, and environmental factors may cause varying responses among individuals to the same exercise intensity (Sun et al., 2024; Cai and Wang, 2024). Future studies should account for these individual differences to better understand how exercise affects T2D risk.

## 5 Strengths and limitations

Although this study explores the potential causal effects of different exercise intensities on type 2 diabetes mellitus (T2D) risk using Mendelian randomization (MR) methods, there are still some limitations to note.

### 5.1 Strengths

#### 5.1.1 Utilization of Mendelian Randomization (MR) method

The MR method leverages genetic variations as instrumental variables, effectively controlling for confounding factors and reverse causation present in traditional observational studies, thereby providing more reliable causal inferences.

#### 5.1.2 Diverse statistical techniques

This study employs multiple robust statistical techniques, including Inverse Variance Weighted (IVW), weighted median, MR-Egger regression, and MR-PRESSO. These methods help to reduce bias from horizontal pleiotropy and other confounding factors, enhancing the reliability of the results.

### 5.2 Limitations

#### 5.2.1 Sample size and statistical power

Although we used data from two large databases, the association between high-intensity exercise and T2D risk did not reach statistical significance. This may be due to an insufficient sample size, leading to a lack of statistical power to detect subtle but true causal effects.

#### 5.2.2 Limitations of genetic instruments

The single nucleotide polymorphisms (SNPs) selected as instrumental variables in this study are limited to known genetic markers associated with exercise intensity, which may omit some important genetic variants. Additionally, the selected SNPs may have unknown horizontal pleiotropy, although we attempted to minimize this impact through various methods.

### 5.3 Future improvements

#### 5.3.1 Increasing sample size

Future studies should increase the sample size, especially including more diverse populations from different races and regions, to improve statistical power and the generalizability of the results.

#### 5.3.2 Integration of multi-omics data

Besides genetic data, integrating epigenomics, transcriptomics, metabolomics, and other multi-omics data will provide a comprehensive analysis of the complex relationship between exercise intensity and T2D risk.

## 6 Conclusion

In summary, increased exercise intensity may reduce the risk of T2D by modulating amino acid metabolism, particularly branched-chain and aromatic amino acids. This finding provides a new perspective on the prevention and treatment of T2D and emphasizes the importance of exercise in the health management of T2D. Future studies should further explore the complex interactions between exercise and T2D pathogenesis in order to develop more precise and effective prevention and treatment strategies.

## Data Availability

The original contributions presented in the study are included in the article/supplementary material, further inquiries can be directed to the corresponding author.
